# Fe_7_Se_8_ encapsulated in N-doped carbon nanofibers as a stable anode material for sodium ion batteries†

**DOI:** 10.1039/d0na00897d

**Published:** 2020-11-10

**Authors:** Le Hu, Chaoqun Shang, Xin Wang, Guofu Zhou

**Affiliations:** Guangdong Provincial Key Laboratory of Optical Information Materials and Technology & Institute of Electronic Paper Displays, South China Academy of Advanced Optoelectronics, South China Normal University Guangzhou 510006 China chaoqun.shang@ecs-scnu.org wangxin@scnu.edu.cn; National Center for International Research on Green Optoelectronics, South China Academy of Advanced Optoelectronics, South China Normal University Guangzhou 510006 China; International Academy of Optoelectronics at Zhaoqing, South China Normal University Zhaoqing 526000 China

## Abstract

Transition metal chalcogenides especially Fe-based selenides for sodium storage have the advantages of high electric conductivity, low cost, abundant active sites, and high theoretical capacity. Herein, we proposed a facile synthesis of Fe_7_Se_8_ embedded in carbon nanofibers (denoted as Fe_7_Se_8_-NCFs). The Fe_7_Se_8_-NCFs with a 1D electron transfer network can facilitate Na^+^ transportation to ensure fast reaction kinetics. Moreover, Fe_7_Se_8_ encapsulated in carbon nanofibers, Fe_7_Se_8_-NCFs, can effectively adapt the volume variation to keep structural integrity during a continuous Na^+^ insertion and extraction process. As a result, Fe_7_Se_8_-NCFs present improved rate performance and remarkable cycling stability for sodium storage. The Fe_7_Se_8_-NCFs exhibit practical feasibility with a reasonable specific capacity of 109 mA h g^−1^ after 200 cycles and a favorable rate capability of 136 mA h g^−1^ at a high rate of 2 A g^−1^ when coupled with Na_3_V_2_(PO_4_)_3_ to assemble full sodium ion batteries.

## Abstract 2

Transition metal chalcogenides especially Fe-based selenides for sodium storage have the advantages of high electric conductivity, low cost, abundant active sites, and high theoretical capacity.

## Graphical abstract

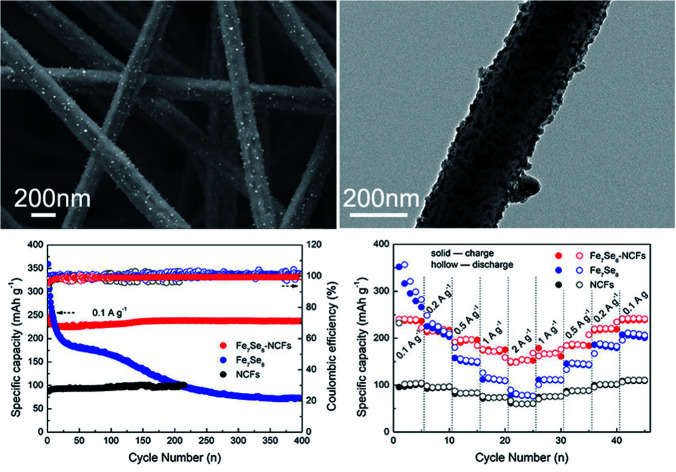

## Introduction

The economical concern of sodium ion batteries (SIBs) has made them one of the large scale energy storage systems to replace lithium ion batteries (LIBs).^1,2^ Besides, SIBs have similar electrochemical storage properties to LIBs, which provides sufficient references for the exploration of electrode materials for SIBs.^3,4^ Graphite as an anode employed in commercial LIBs exhibits undesirable sodium storage capability in SIBs with traditional carbonate-based electrolytes.^5,6^ Therefore, the research of SIB anode materials is still a great challenge compared to the great progress of cathode materials with promising electrochemical performance.^7,8^

Over the past few years, tremendous efforts have been made to search for appropriate anode materials of SIBs, such as hard or soft carbon, alloy-type Sn/Sb/Bi, transition metal oxides/sulfides/phosphides/selenides, organic molecules, *etc.*^9,10^ As potential candidates, transition metal selenides especially Fe-based selenides have the advantages of high electric conductivity, low cost, abundant active sites, and high theoretical capacity.^11,12^ However, the large radius of sodium ions results in sluggish reaction kinetics and large volume variation during sodium ion insertion/deinsertion and further hinders the rate capability and cycling stability of the corresponding SIBs.^13,14^ To address these issues, downsizing the active materials to the nanoscale and surface coating to suppress the volume effect are common strategies.^15,16^ For example, Chen *et al.* reported that micro-nano hierarchitectures of urchin-like Fe_3_Se_4_ effectively display remarkable rate performance (200.2 mA h g^−1^ at 30 A g^−1^).^17^ Li and coworkers studied FeSe nanoparticles embedded in N-doped carbon (FeSe/N–C) with stable cycling performance (333.9 mA h g^−1^ after 800 cycles).^18^ Nevertheless, further improvement of the sodium storage capability of Fe-based selenides is still a challenge.

In this paper, we proposed a facile synthesis of Fe_7_Se_8_ embedded in carbon nanofibers (denoted as Fe_7_Se_8_-NCFs), which was further assembled as an anode material for SIBs. Taking advantage of downsized dimensions, effective carbon buffer protection, and the 1D nanofiber electron transfer network, Fe_7_Se_8_-NCFs shows remarkable stability (232 mA h g^−1^ at 0.1 A g^−1^, 400 cycles) and improved rate capability (153 mA h g^−1^, 2 A g^−1^) in half-cells. The electrochemical kinetic analysis unveils that the surface pseudocapacitive process contributes largely to the capacity, which undoubtedly enhances the rate capability. Moreover, Fe_7_Se_8_-NCFs coupled with Na_3_V_2_(PO_4_)_3_ (denoted as NVP) also shows potential in full SIBs, which display favorable rate capability and reasonable reversible capacity.

## Experimental section

### Preparation of Fe_7_Se_8_-NCFs nanofibers

Polyacrylonitrile (PAN, 0.7 g) and iron(iii) acetylacetonate (Fe(AcAc)_3_, 0.3 g) were mixed in dimethylformamide (DMF, 10 ml) by constantly stirring for 10 h to obtain a uniformly dispersed solution. Then, the as-prepared solution was put into a syringe and electrospun at a flow rate of 0.1 mm min^−1^ at high voltage (14 kV). The obtained Fe(AcAc)_3_/PAN nanofibers (0.1 g) and Se powder (0.2 g) were annealed at a temperature of 500 °C for 4 h (H_2_/Ar). For the comparative sample, Fe_7_Se_8_ powder was prepared by the same annealing process using Fe(AcAc)_3_ and Se powder (weight ratio: 1 : 2). The N-doped carbon nanofibers (NCFs) were annealed by the same process using PAN nanofibers. NVP was prepared following a previous report.^19^ NaH_2_PO_4_ (0.85 g), NH_4_VO_3_ (0.47 g) and citric acid (0.77 g) were dissolved in deionized water (60 ml) with continuous agitation. Graphene oxide (0.04 g) was mixed into the above solution and then freeze-dried for 24 h. The as-prepared powder was calcined at 800 °C for 8 h (Ar/H_2_) to obtain NVP.^20^ The material characterization section is in the ESI.†

### Electrochemical measurement

A slurry was prepared by mixing 80 wt% active material, 10 wt% Super P, and 10 wt% binder (polyvinylidene fluoride, PVDF) and was cast onto Cu foil and dried in a vacuum at 60 °C for 12 h to prepare the working electrode. The working electrode was cut into discs with a diameter of 12 mm. The full cell consists of the Fe_7_Se_8_-NCF anode and NVP cathode. The NVP cathode was prepared by mixing NVP, Super P and polytetrafluoroethylene (PTFE) (weight ratio: 8 : 1 : 1). For the NVP‖Fe_7_Se_8_-NCFs full cell, the mass ratio of the cathode and anode was controlled at 5 : 1. The electrolyte was NaClO_4_ (1 M) dissolved in a mixture of ethylene carbonate and dimethyl carbonate (volume ratio: EC/DMC = 1/1) solution with fluoroethylene carbonate (FEC: 5 vol%). Electrochemical impedance spectroscopy (EIS) measurements were performed in the frequency range from 100 kHz to 100 mHz. Cyclic voltammetry (CV) was performed on a BioLogic (VMP3) electrochemical workstation in the voltage range of 0.1–3 V.

## Results and discussion

**Fig. 1:**
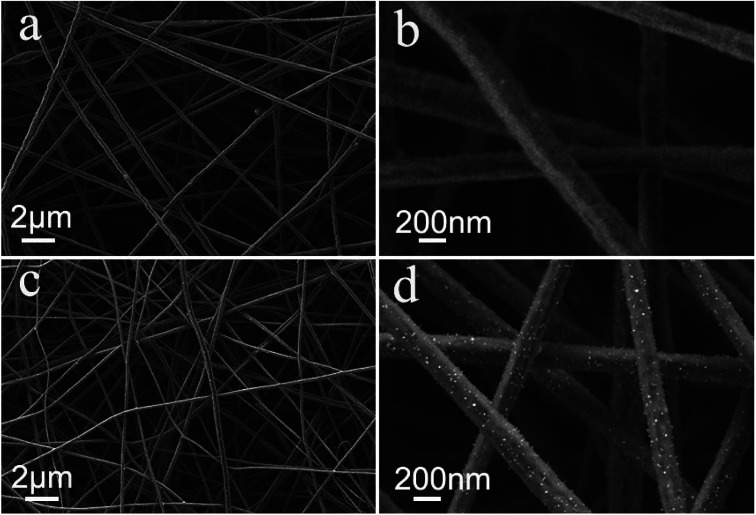
(a and b) SEM images of Fe(AcAc)_3_/PAN nanofibers; (c and d) SEM images of as-prepared Fe_7_Se_8_-NCFs.

**Fig. 2:**
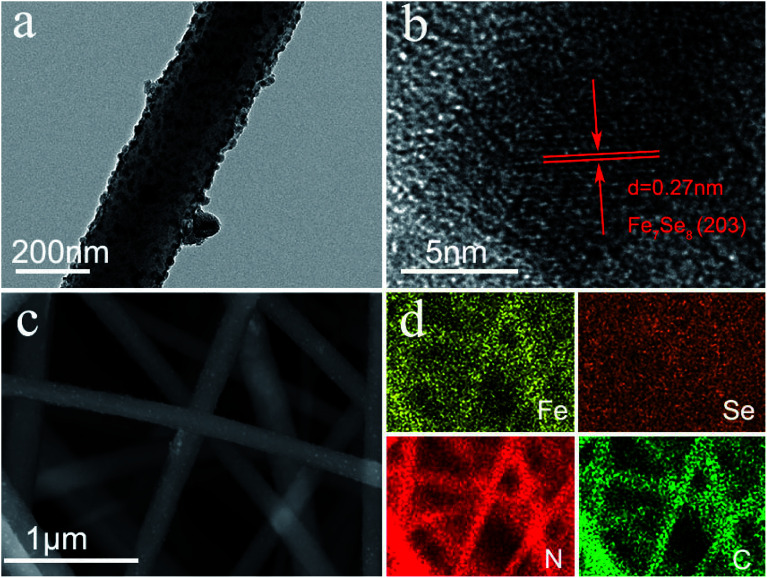
Fe_7_Se_8_-NCFs: (a) TEM; (b) HRTEM; (c) SEM image and (d) corresponding EDS mapping.

The synthesis process of the Fe_7_Se_8_-NCFs nanofibers is mainly divided into electrospinning and subsequent selenization processes (Fig. S1†). The SEM image of as-electrospun Fe(AcAc)_3_/PAN shows smooth and evenly distributed nanofibers, as exhibited in Fig. 1a and b. After the selenization process, Fe_7_Se_8_-NCFs well maintains the 1D fiber structure of Fe(AcAc)_3_/PAN and regular distribution (Fig. 1c and d). The cross-sectional SEM images of Fe_7_Se_8_-NCFs show abundant voids between the nanofibers, which facilitate the penetration of the electrolyte (Fig. S2†). Combined with SEM morphologies, the TEM image further reveals that the diameter of Fe_7_Se_8_-NCFs nanofibers is about 200 nm, and the Fe_7_Se_8_ nanoparticles are uniformly embedded in N-doped carbon nanofibers (Fig. 2a). The high-resolution TEM image (HRTEM, Fig. 2b) of Fe_7_Se_8_-NCFs shows a lattice fringe of 0.27 nm, indexed to the (203) plane of the Fe_7_Se_8_ phase. The SEM image and energy dispersive spectroscopy (EDS) elemental mappings reveal Fe, Se, N and C elements uniformly distributed in the carbon nanofibers (Fig. 2c and d). However, in Fig. S3,† the as-prepared Fe_7_Se_8_ bulk is composed of large aggregated particles due to lack of a fiber support.

**Fig. 3:**
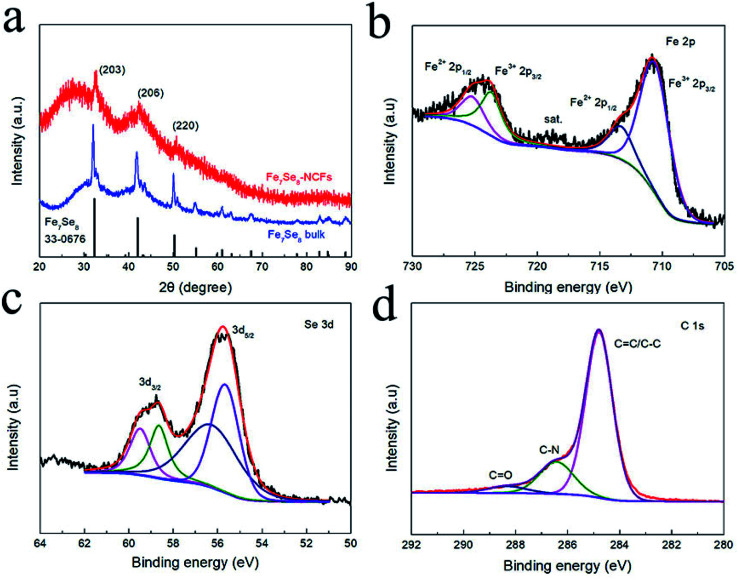
(a) XRD pattern of Fe_7_Se_8_-NCFs. High-resolution XPS spectra of Fe_7_Se_8_-NCFs: (b) Fe 2p; (c) Se 3d; (d) C 1s.

Fig. 3a displays the XRD pattern of Fe_7_Se_8_-NCFs and bulk Fe_7_Se_8_. The major diffraction peaks at 32.3, 42.0 and 50.2° are assigned to the (203), (206) and (220) planes of Fe_7_Se_8_ (PDF#33-0676). It should be noted that the XRD peaks of Fe_7_Se_8_-NCFs slightly shift to high 2*θ*. This indicates the existence of Se vacancies owing to the protection of the NCF substrate during selenization, which can enhance the electronic conductivity, guaranteeing fast electron transfer for Na^+^ insertion/extraction. The diffraction peaks of Fe_7_Se_8_-NCFs are broader than those of bulk Fe_7_Se_8_, which demonstrates the small crystal size of Fe_7_Se_8_ in the NCF matrix. This is beneficial to shorten the sodium ion diffusion length during sodium ion insertion and deinsertion. According to the Scherrer formula (the details are in the ESI†), the crystal size of Fe_7_Se_8_ in NCFs is ∼20 nm, while that of bulk Fe_7_Se_8_ is 40 nm. The Fe_7_Se_8_ content in the Fe_7_Se_8_-NCF composite was determined by a TGA test (Fig. S4a†). The mass loss below 200 °C is due to the evaporation of the residual moisture in the Fe_7_Se_8_-NCF sample. The temperature between 300 and 500 °C can be assigned to the oxidation of carbon fibers and Fe_7_Se_8_. When the temperature reaches 600 °C in air, the final combustion product of Fe_7_Se_8_-NCFs is Fe_2_O_3_ (Fig. S4b†).^21^ Thus, the content of Fe_7_Se_8_ in dried Fe_7_Se_8_-NCFs is ∼20.6%. An XPS test was performed to analyze the chemical composition of Fe_7_Se_8_-NCFs. The survey spectrum of Fe_7_Se_8_-NCFs illustrates the coexistence of Fe, Se, N and C elements in Fe_7_Se_8_-NCFs (Fig. S5a†). As presented in Fig. 3b, the Fe 2p spectrum can be divided into Fe^2+^ and Fe^3+^ for the spin–orbit doublet of Fe 2p_1/2_ and Fe 2p_3/2_. The peaks at 725.2 and 713.5 eV are assigned to 2p_1/2_ and 2p_3/2_ of Fe^2+^, and the peaks at 723.7 and 710.6 eV are ascribed to 2p_1/2_ and 2p_3/2_ of Fe^3+^, demonstrating the presence of Fe^2+^ and Fe^3+^ states in Fe_7_Se_8_-NCFs.^22^ In Fig. 3c, the XPS spectrum of Se 3d consists of Se 3d_3/2_ (59.5 and 58.6 eV) and 3d_5/2_ (56.3 and 55.7 eV).^23^ In Fig. 3d, the C 1s spectra displays three peaks located at 288.4 eV (C=O), 286.4 eV (C–N), and 284.8 eV (C–C/C=C) respectively.^24^ The N-doping carbon layer not only enhances electronic conductivity to promote fast reaction kinetics, but also relieves volume variation to keep the stable structure of Fe_7_Se_8_-NCFs during repeated cycling. The XPS spectrum and detailed analysis of other elements (O and N) are exhibited in Fig. S5.† In the Raman analysis, the peaks at about 278, 330, and 405 cm^−1^ might be assigned to Fe–Se bonds in Fe_7_Se_8_-NCFs, and other peaks at 1358 cm^−1^ and 1589 cm^−1^ are ascribed to the D band and G band, respectively^25^ (Fig. S6†). The *I*_D_/*I*_G_ of Fe_7_Se_8_-NCFs (1.76) and NCFs (1.64) indicates more defects of carbon on Fe_7_Se_8_-NCFs to promote reaction kinetics.

**Fig. 4:**
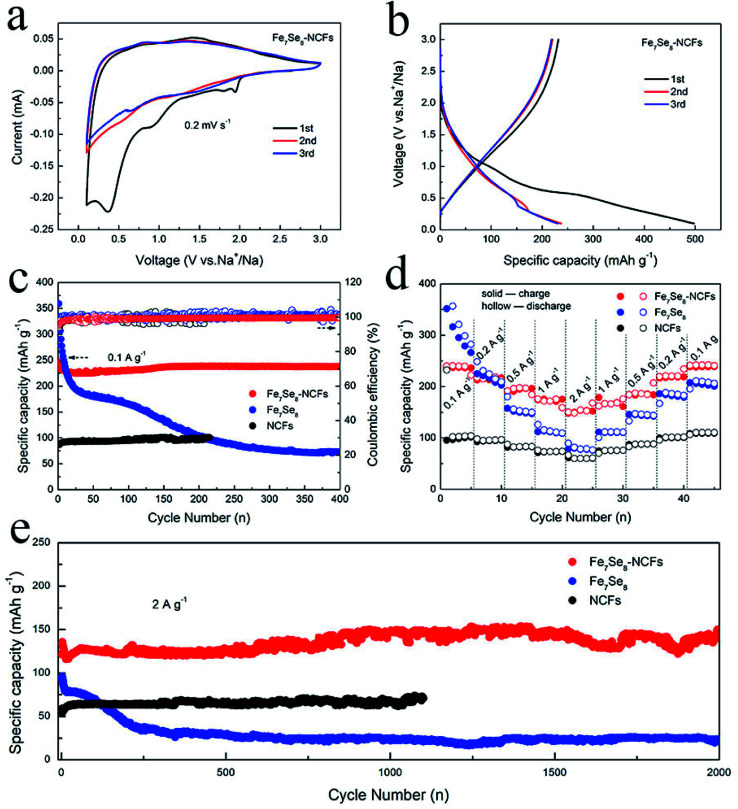
(a) CV curves of Fe_7_Se_8_-NCFs and (b) charge/discharge curves of Fe_7_Se_8_-NCFs. Electrochemical performance of Fe_7_Se_8_-NCFs, Fe_7_Se_8_ and NCFs: (c) cycling (0.1 A g^−1^); (d) rate; and (e) cycling performance (2 A g^−1^).

The electrochemical performance in sodium storage of Fe_7_Se_8_-NCFs was investigated by CV measurements (Fig. 4a). In the initial cycle, the sharp reduction peaks at 0.4 and 0.9 V originate from the generation of a solid electrolyte interface (SEI) film and irreversible Na^+^ insertion.^26,27^ In the following curves, the broad redox peaks at 1.6 and 0.9 V are well overlapped, indicating a reversible conversion reaction for Fe_7_Se_8_-NCFs. The redox peaks of the CV curves are not obvious, which might be due to the carbon effect in Fe_7_Se_8_-NCFs. The 1D carbon fiber as a buffer matrix maintains the structural integrity to guarantee a long cycle life. In Fig. 4b, the charge/discharge profiles of Fe_7_Se_8_-NCFs show that the initial charge/discharge specific capacity is 232/497 mA h g^−1^ and the corresponding initial coulombic efficiency (ICE) is 46.7%. The low ICE might be ascribed to solid electrolyte interface (SEI) formation and some irreversible side reactions, which are consistent with the CV result.^28^Fig. 4c depicts the cycling performance of Fe_7_Se_8_-NCFs compared with those of Fe_7_Se_8_ and NCFs. When the current density is 0.1 A g^−1^, Fe_7_Se_8_-NCFs delivers a reversible capacity of 223 mA h g^−1^ after 400 cycles with a capacity retention rate of 96.5%, providing an impressive CE of around 100%. The comparative Fe_7_Se_8_ displays a rapid decrease in current density from 359 to 73 mA h g^−1^ during the 400 cycles. The high initial specific capacity of comparative Fe_7_Se_8_ is due to the high theoretical specific capacity of the Fe_7_Se_8_ active material (419 mA h g^−1^). However, Fe_7_Se_8_ undergoes serious volume change during larger Na^+^ intercalation and deintercalation, which causes the active material to be pulverized with continuous capacity degradation. With the protection of NCFs, Fe_7_Se_8_-NCFs could adapt to volume expansion to retain a stable capacity. The rate capability of Fe_7_Se_8_-NCFs, Fe_7_Se_8_ and NCFs is further tested and compared, as exhibited in Fig. 4d. As the current density increases from 0.1 to 2 A g^−1^, Fe_7_Se_8_-NCFs delivers a specific capacity of 237, 215, 195, 175 and 153 mA h g^−1^. Impressively, when the current density is switched back to 0.1 A g^−1^, Fe_7_Se_8_-NCFs recovers a specific capacity of 238 mA h g^−1^ without any attenuation, demonstrating favorable rate performance of Fe_7_Se_8_-NCFs. In contrast, Fe_7_Se_8_ shows poor rate performance (from 357 to 70 mA h g^−1^ at 0.1 to 2 A g^−1^). The promising rate performance of Fe_7_Se_8_-NCFs could be attributed to the 1D carbon nanofibers, which can shorten the ion diffusion pathway to facilitate Na^+^ transportation. As shown in Table S1,† the research of Fe_7_Se_8_-NCFs for sodium storage exhibits stable electrochemical performance.^29^ Furthermore, Fig. 4e shows the long-term cycling stability of Fe_7_Se_8_-NCFs with a specific capacity of 148 mA h g^−1^ after 2000 cycles, which is more stable than those of Fe_7_Se_8_ and NCFs (the current density: 2 A g^−1^). These results further reveal the strong structural stability of Fe_7_Se_8_ encapsulated in 1D NCFs.

**Fig. 5:**
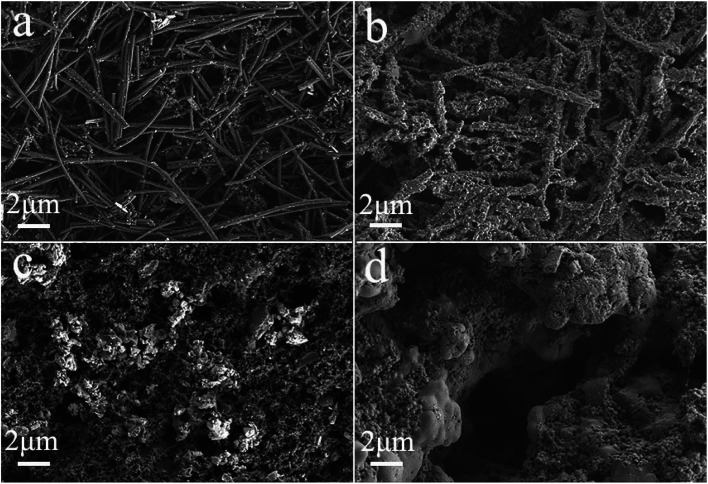
SEM images of Fe_7_Se_8_-NCFs and Fe_7_Se_8_: (a and c) before cycling; (b and d) after 100 cycles in the charged state, respectively.

To deeply elucidate the stable carbon matrix structure of Fe_7_Se_8_-NCFs, the surface morphology of Fe_7_Se_8_-NCFs was observed before and after 100 cycles. The SEM morphology of fresh Fe_7_Se_8_-NCFs with regular nanofibers is shown in Fig. 5a. The cycled Fe_7_Se_8_-NCFs retains a flat and distinct 1D nanofiber structure with abundant voids (Fig. 5b), which is similar to the original shape. Meanwhile, the slight volume expansion of cycled Fe_7_Se_8_-NCFs might be due to the generation of a stable SEI film. These results suggest that the Fe_7_Se_8_ embedded in a 1D carbon matrix can effectively alleviate the volume variation and maintain the structural integrity to guarantee a long cycle life. Additionally, the SEM image of fresh Fe_7_Se_8_ has large bulk particles, as shown in Fig. 5c. After 100 cycles, Fe_7_Se_8_ showed serious volume expansion compared with the pristine appearance (Fig. 5d), which is in accordance with the continuous capacity deterioration of Fe_7_Se_8_ during cycling (Fig. 4c).

**Fig. 6:**
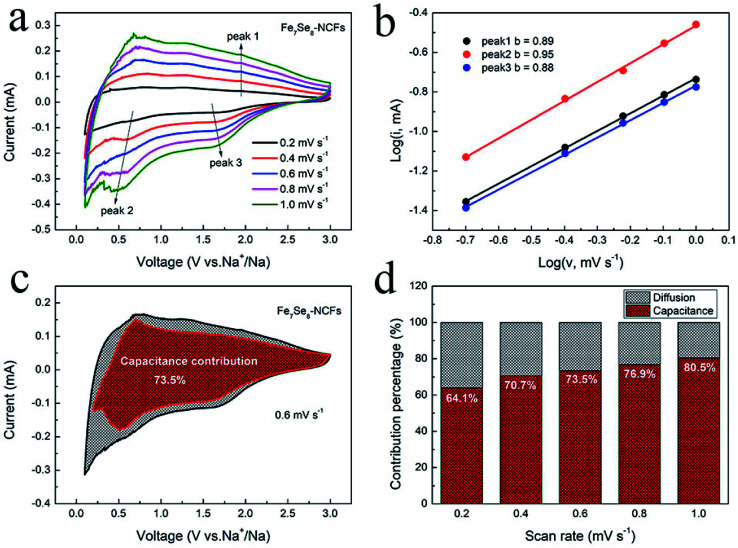
Kinetics analysis of Fe_7_Se_8_-NCFs: (a) CV curves; (b) the corresponding log(*i*) *versus* log(*v*) plots; (c) capacitive and diffusion-controlled contribution (0.6 mV s^−1^); (d) ratio of pseudocapacitive contribution.

Fig. 6 displays the kinetics analysis of Fe_7_Se_8_-NCFs to understand the promising rate performance in sodium storage. When the scan rate increases from 0.2 to 1 mV s^−1^, the CV curves of Fe_7_Se_8_-NCFs and Fe_7_Se_8_ show a similar shape (Fig. 6a and S7a†). The current (*i*) and scan rate (*v*) conform to the equation as follows:^30,31^

1 *i* = *av*^*b*^

2 log(*i*) = log(*a*) + *b* log(*v*)

The *b* value is the slope of eqn (2), which is in the range from 0.5 to 1. In general, a *b* value of 0.5 or 1 represents a capacitive-controlled or diffusion-controlled process. For Fe_7_Se_8_-NCFs, the *b* value of oxidation and reduction peaks is calculated to be 0.89, 0.95 and 0.88, higher than those of Fe_7_Se_8_ (0.51, 0.51 and 0.55) (Fig. 6b and S7b†). All *b* values of Fe_7_Se_8_-NCFs are close to 1, which suggests mainly pseudocapacitive behavior in redox reactions. On the other hand, we calculated the ratio of pseudocapacitive contribution according to the following equation:^32,33^

3 *i* = *k*_1_*υ* + *k*_2_*υ*^1/2^

4 *i*/*υ*^1/2^ = *k*_1_*υ*^1/2^ + *k*_2_

Here, *k*_1_*υ* and *k*_2_*υ*^1/2^ represent the capacitive contribution and ion-diffusion contribution, respectively. Fig. 6c displays the proportion of capacitive contribution of Fe_7_Se_8_-NCFs at 0.6 mV s^−1^ (73.5%), which is much higher than that of Fe_7_Se_8_ (32.5%, Fig. S7c†). The incorporation of Fe_7_Se_8_ inti a 1D carbon fiber could obviously increase percentage of capacitive contributions to facilitate fast reaction kinetics for Fe_7_Se_8_-NCFs. In Fig. 6d, we summarized the proportion of surface pseudocapacitive contribution from 0.2 mV s^−1^ to 1 mV s^−1^. When the sweep rate increases, the pseudocapacitive contribution of Fe_7_Se_8_-NCFs gradually increases, reaching as high as 80.5% at 1 mV s^−1^. The high percentage of surface pseudocapacitive contribution is beneficial to fast electron/ion transport kinetics to contribute to the high-rate performance of Fe_7_Se_8_-NCFs. EIS was conducted to analyze the change in the charge-transfer resistance (*R*_ct_) of Fe_7_Se_8_-NCFs before and after 100 cycles. From the fitting equivalent circuit, the *R*_ct_ of Fe_7_Se_8_-NCFs in the pristine state is ∼223 Ω. As the cycle number increases to 100, the *R*_ct_ of cycled Fe_7_Se_8_-NCFs decreases to ∼169 Ω (Fig. S8†). The decline in *R*_ct_ of Fe_7_Se_8_-NCFs after cycling could be attributed to the activation process during cycling, thus enhancing the charge transfer process and kinetics reaction.^34^

**Fig. 7:**
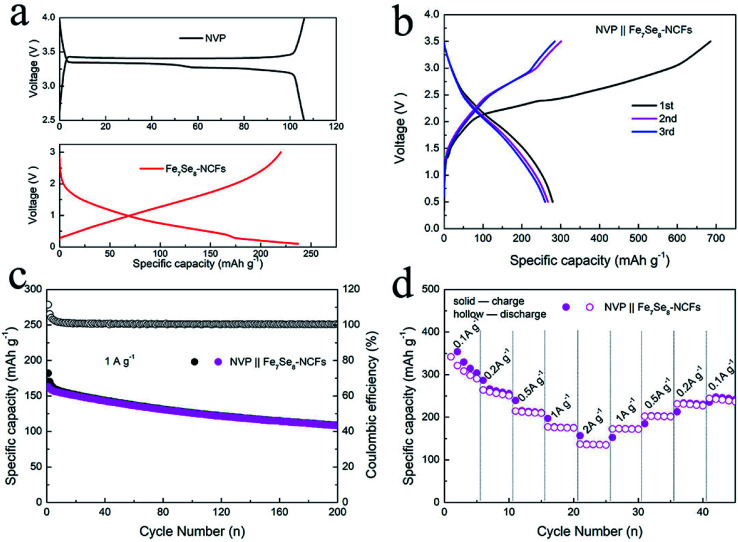
(a) Charge/discharge curves of NVP and Fe_7_Se_8_-NCF electrodes in a half-cell. (b) Charge/discharge curves, and (c) cycling and (d) rate performance of the NVP‖Fe_7_Se_8_-NCF full cell.

In view of the impressive performance of Fe_7_Se_8_-NCFs in half cells, a sodium ion full cell was fabricated to explore its practical feasibility. The Fe_7_Se_8_-NCF anode was coupled with an excessive NVP cathode to assemble the full cell. The SEM morphology, the XRD pattern and sodium storage performance of NVP are provided in Fig. S9.† The charge/discharge curves of NVP display a voltage plateau of 3.3 V in the half cell (Fig. 7a). The NVP in the half cell delivers a stable specific capacity of 99 mA h g^−1^ after 100 cycles and a favorable rate performance of 81 mA h g^−1^ at a high rate of 2 A g^−1^, which is in good agreement with previous research.^35^ Based on the weight of the Fe_7_Se_8_-NCF anode, we examined the electrochemical performance of the NVP‖Fe_7_Se_8_-NCF sodium ion full cell. The charge/discharge curves of the NVP‖Fe_7_Se_8_-NCF full cell between 0.5 and 3.5 V are obtained, which exhibits an initial charge/discharge capacity of 685/279 mA h g^−1^ with an ICE of 40.7% (Fig. 7b). The low ICE may be attributed to the formation of a SEI film and some irreversible reactions.^36^ The cycling and rate capability of the NVP‖Fe_7_Se_8_-NCF full cell are evaluated as shown in Fig. 7c and d. The specific capacity of the full cell remains at 109 mA h g^−1^ at 1 A g^−1^ with a CE of ∼100% after 200 cycles. Moreover, the NVP‖Fe_7_Se_8_-NCF full cell exhibits favorable rate performance with a specific capacity of 136 mA h g^−1^ at 2 A g^−1^. These results demonstrate the application potential of Fe_7_Se_8_-NCFs in sodium ion batteries.

## Conclusion

In conclusion, unique Fe_7_Se_8_ embedded in carbon nanofibers was successfully designed for sodium storage. In consideration of the 1D carbon nanofiber network, Fe_7_Se_8_-NCFs can facilitate Na^+^ transportation by increasing the contact area between the electrode and electrolyte to promote electrochemical reaction kinetics. Moreover, Fe_7_Se_8_ encapsulated in carbon nanofibers, the Fe_7_Se_8_-NCF electrode, can effectively accommodate the volume change to keep the structural integrity of Fe_7_Se_8_-NCFs during a conversion reaction process. Hence, the Fe_7_Se_8_-NCF electrode shows remarkable stability (223 mA h g^−1^ at 0.1 A g^−1^, after 400 cycles) and improved rate capability (153 mA h g^−1^, 2 A g^−1^) in half-cells. Impressively, when coupled with NVP to assemble a full cell, the NVP‖Fe_7_Se_8_-NCF full cell exhibits its practical feasibility with a specific capacity of 109 mA h g^−1^ after 200 cycles (1 A g^−1^) and a favorable rate capability of 136 mA h g^−1^ at a high rate of 2 A g^−1^.

## Conflicts of interest

There are no conflicts to declare.

## Supplementary Material

NA-003-D0NA00897D-s001
