# Supplementary material for: Fe7Se8 encapsulated in N-doped carbon nanofibers as a stable anode material for sodium ion batteries
Source: Nanoscale Adv. 2020 Nov 10;3(1):231–9. doi: 10.1039/d0na00897d (PMC9419117; doi:10.1039/d0na00897d)
Supplement: NA-003-D0NA00897D-s001 [file NA-003-D0NA00897D-s001.pdf]

Electronic Supplementary Material (ESI) for Nanoscale Advances.
This journal is © The Royal Society of Chemistry 2020

Fe_7Se_8 Encapsulated in N-doped Carbon Nanofibers as Stable Anode Materials for Sodium Ion Batteries

Le Hu[1#], Chaoqun Shang[1#] *, Xin Wang[1,2,3] *, Guofu Zhou[1,2,3]

[1] Guangdong Provincial Key Laboratory of Optical Information Materials and Technology & Institute of Electronic Paper Displays, South China Academy of Advanced Optoelectronics, South China Normal University, Guangzhou 510006, China

[2] National Center for International Research on Green Optoelectronics, South China Academy of Advanced Optoelectronics, South China Normal University, Guangzhou 510006, China

[3] International Academy of Optoelectronics at Zhaoqing, South China Normal University, Zhaoqing 526000, China

[#]These authors contributed equally.

*Corresponding author:

chaoqun.shang@ecs-scnu.org

wangxin@scnu.edu.cn

Material Characterization

The morphology of all the samples was observed by a field emission scanning electron microscope (FESEM, Zeiss Ultra 55) and a transmission electron microscope (TEM, JEOL JEM-2100F). Thermal gravimetric analysis (TGA, SDT Q600 TG-DTA) was carried out to confirm the content of Fe_7Se_8. The crystal phase was investigated by x-ray diffraction (XRD, Bruker D8 advance). The surface chemical compositions were measured on an x-ray photoelectron spectrometer (XPS, Kratos AXIS Ultra DLD).

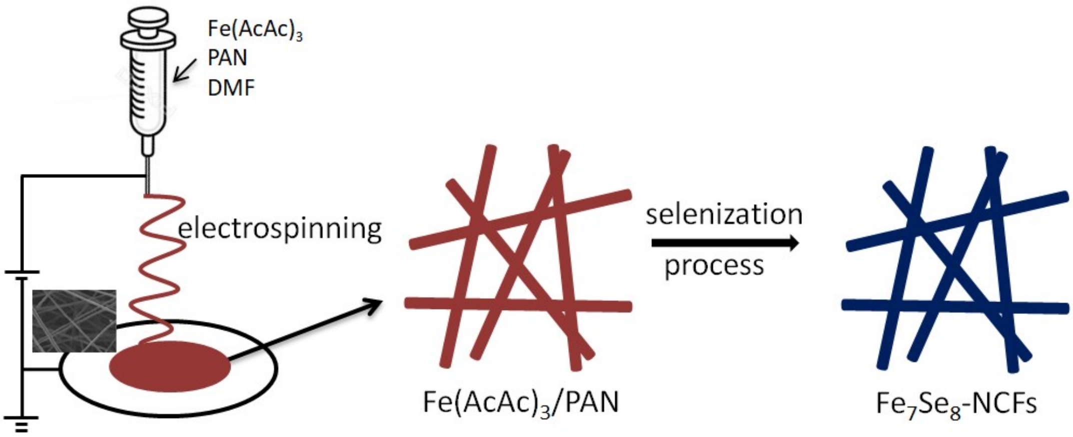

Figure S1. The schematic synthesis process of Fe_7Se_8-NCFs nanofibers.

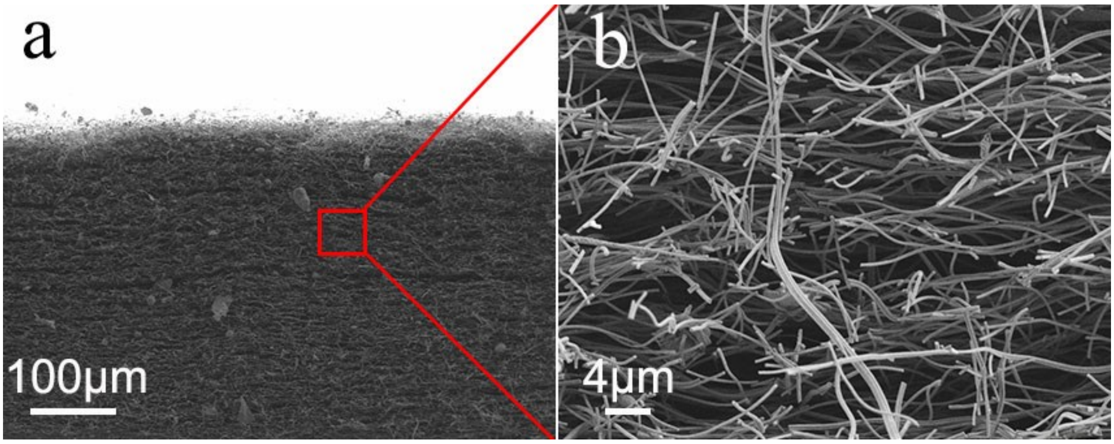

Figure S2. Cross-section SEM images of Fe_7Se_8-NCFs nanofibers.

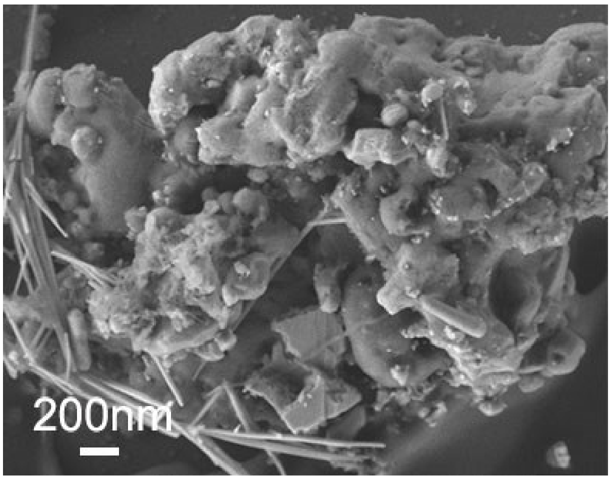

Figure S3. SEM image of bulk Fe_7Se_8.

The crystal size is calculated by the Scherrer's equation as follows:

$$D = \frac{K\lambda}{\beta \cos\theta}$$

Here, D is the average crystal diameter, K is a constant related the shape of the crystallites, λ is the wavelength of the X-rays employed, β is the corrected peak width (full width at half-maximum) and θ is the diffraction angle.

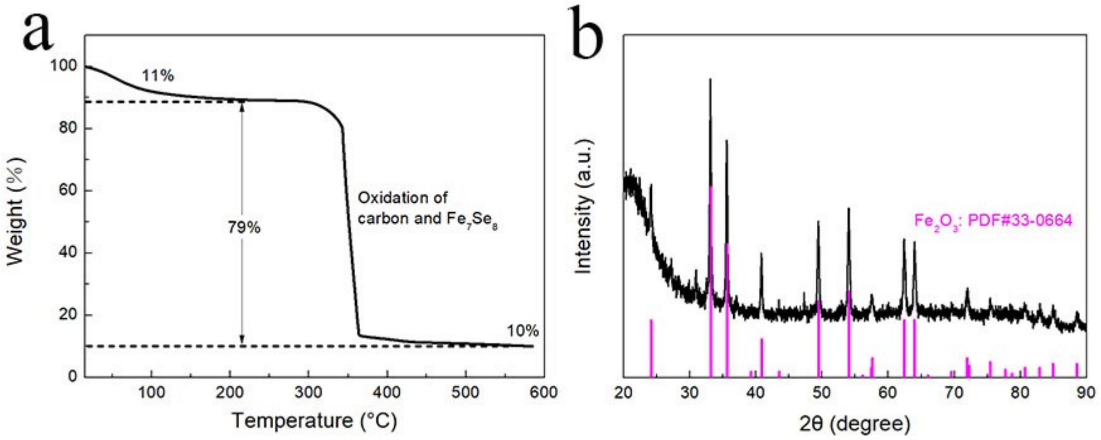

Figure S4. (a) the TGA spectra of Fe_7Se_8-NCFs; (b) the combustion product of Fe_7Se_8-NCFs.

The calculation of Fe_7Se_8 content in Fe_7Se_8-NCFs:

$4Fe_7Se_8 + 53O_2 = 14Fe_2O_3 + 32SeO_2$

$$Fe_7Se_8(wt\%) = \frac{4 \times molecular\ weight\ of\ Fe_7Se_8}{14 \times molecular\ weight\ of\ Fe_2O_3} \times \frac{weight\ of\ Fe_2O_3}{weight\ of\ Fe_7Se_8 - NC} \times 100\%$$

$$= \frac{4 \times 1022.6}{14 \times 159.7} \times \frac{1 - 0.79 - 0.11}{1 - 0.11} \times 100\%$$

$$= 20.6\%$$

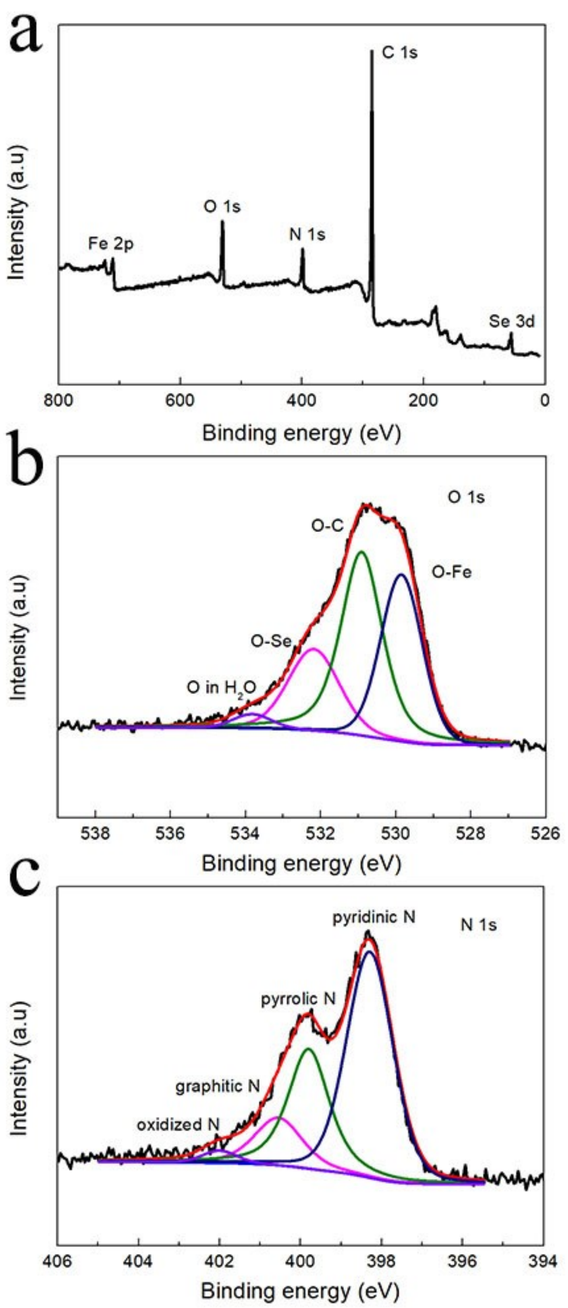

Figure S5. XPS survey spectra and corresponding (b) O 1s and (c) N 1s and (d) C 1s of the Fe_7Se_8-NCFs, respectively.

The O 1s spectrum is convoluted into four peaks at 533.8, 532.2, 530.9 and 529.8 eV, which are attributed to adsorbed water on the surface of sample, Se-O, C-O and Fe-O bonds (Figure S5b)[1]. The N 1s spectrum is deconvoluted into representative peaks centered at 402, 400.5, 399.8 and 398.3 eV, attributing to oxidized-N, graphitic-N, pyrrolic-N and pyridinic-N (Figure S5c)[2]

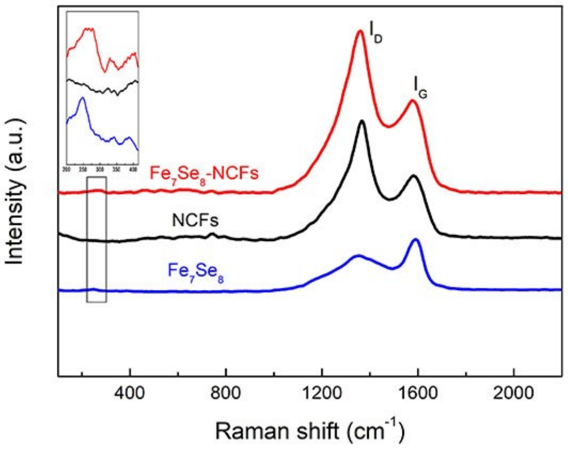

.

Figure S6. Raman spectra of Fe_7Se_8-NCFs, Fe_7Se_8 and NCFs, respectively.

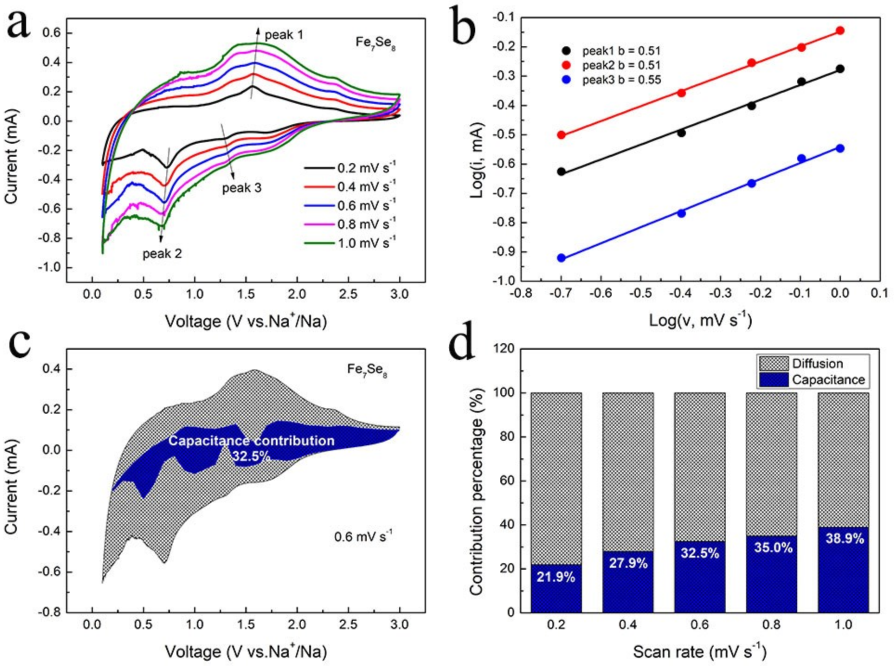

Figure S7. Kinetic analysis for sodium storage: (a) CV curves of Fe_7Se_8 at different scan rates; (b) fitting line of log(i) versus log(v) plots of MnSe; (c) capacitive and diffusion-controlled contribution of Fe_7Se_8 at 0.6 mV s^{-1}; (d) ratio of contribution capacitive of Fe_7Se_8 at different scan rates.

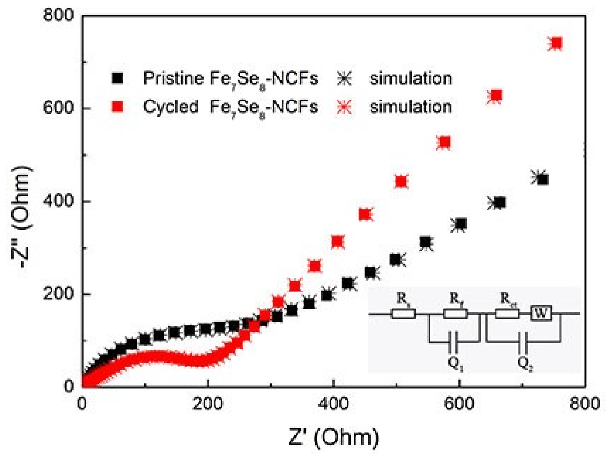

Figure S8. EIS curves at pristine and cycled state of Fe_7Se_8-NCFs.

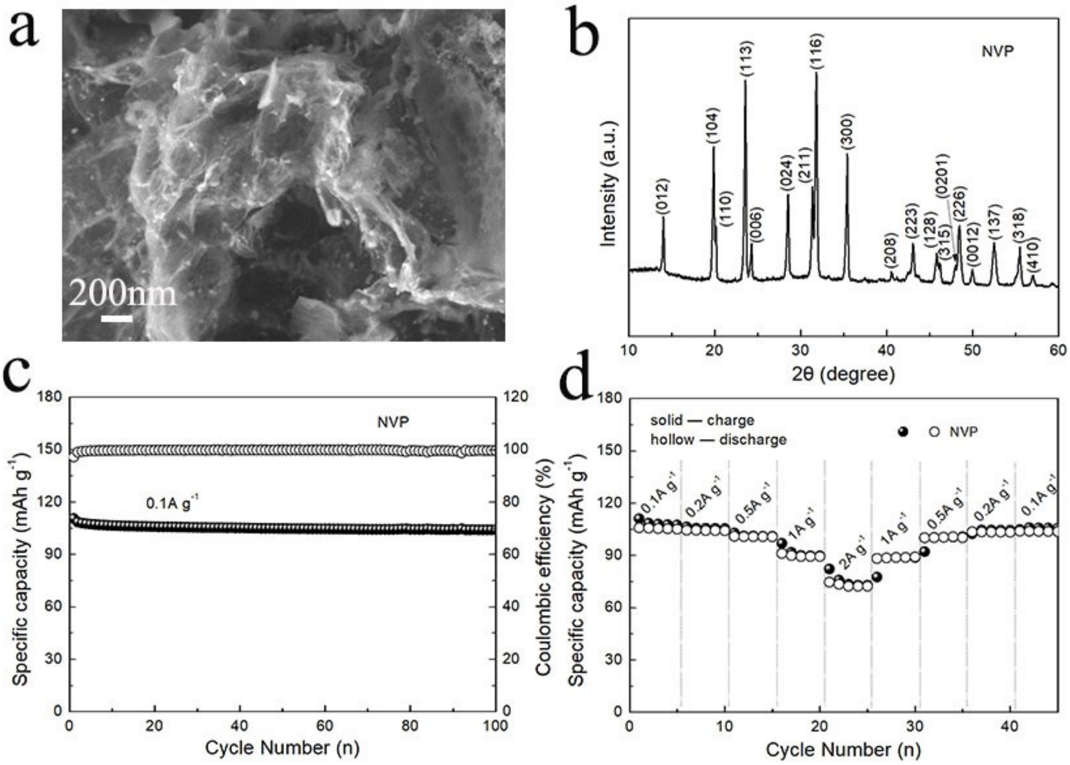

Figure S9. (a) SEM image and (b) XRD pattern of the NVP. (c) Cycling performance and (d) rate capability of the NVP cathode between 2.5 and 4.0 V in half cell.

Table S1. The comparison of electrochemical performance for Fe_7Se_8-NCFs and other Fe_7Se_8-based electrodes in sodium storage.

Samples	Sodium ion batteries	
	Cycling performance (reversible capacity)	Rate performance
Fe_7Se_8-NCFs (This work)	148 mAh g^{-1} at 2 A g^{-1} (2000 cycles)	153 mAh g^{-1} at 2 A g^{-1}
Fe_7Se_8/N–CNF (Ref. 12)	405.6 mAh g^{-1} at 0.1 A g^{-1} (500 cycles)	364 mAh g^{-1} at 2 A g^{-1}
Fe_7Se_8@C/N NBs (Ref. 14)	385 mAh g^{-1} at 0.1 A g^{-1} (350 cycles)	320 mAh g^{-1} at 2 A g^{-1}
Fe_7Se_8@C (Ref. 21)	399 mAh g^{-1} at 0.5 A g^{-1} (150 cycles)	353 mAh g^{-1} at 2 A g^{-1}
Fe_7Se_8@NC (Ref. 25)	367 mAh g^{-1} at 0.5 A g^{-1} (400 cycles)	251 mAh g^{-1} at 2 A g^{-1}
Fe_7Se_8 NRBs (Ref. 29)	300 mAh g^{-1} at 0.5 A g^{-1} (500 cycles)	245 mAh g^{-1} at 2 A g^{-1}

Reference:

[1] C. Yuan, J. Li, L. Hou, X. Zhang, L. Shen, X.W.D. Lou, Ultrathin Mesoporous $NiCo_2O_4$ Nanosheets Supported on Ni Foam as Advanced Electrodes for Supercapacitors, Adv. Funct. Mater, 22 (2012) 4592-4597.

[2] Q. Zhao, R. Bi, J. Cui, X. Yang, L. Zhang, TiO_{2-x} Nanocages Anchored in N-Doped Carbon Fiber Films as a Flexible Anode for High-Energy Sodium-Ion Batteries, ACS Appl. Energy Mater., 1 (2018) 4459-4466.
